# Comparative analysis of bevacizumab and LITT for treating radiation necrosis in previously radiated CNS neoplasms: a systematic review and meta-analysis

**DOI:** 10.1007/s11060-024-04650-1

**Published:** 2024-04-15

**Authors:** Neslihan Nisa Gecici, Muhammet Enes Gurses, Brandon Kaye, Natasha L. Frontera Jimenez, Chandler Berke, Elif Gökalp, Victor M. Lu, Michael E. Ivan, Ricardo J. Komotar, Ashish H. Shah

**Affiliations:** 1 Department of Neurological Surgery, University of Miami Miller School of Medicine, Miami, FL 33136 US; 2 Dr. Kiran C. Patel College of Allopathic Medicine, Davie, FL 33326 US; 3 Department of Neurosurgery, University of Puerto Rico School of Medicine, San Juan, Puerto Rico

**Keywords:** Bevacizumab, Laser interstitial thermal therapy, Radiation necrosis, Radiotherapy, Stereotactic radiosurgery

## Abstract

**Purpose:**

Radiation necrosis (RN) is a local inflammatory reaction that arises in response to radiation injury and may cause significant morbidity. This study aims to evaluate and compare the efficacy of bevacizumab and laser interstitial thermal therapy (LITT) in treating RN in patients with previously radiated central nervous system (CNS) neoplasms.

**Methods:**

PubMed, Cochrane, Scopus, and EMBASE databases were screened. Studies of patients with radiation necrosis from primary or secondary brain tumors were included. Indirect meta-analysis with random-effect modeling was performed to compare clinical and radiological outcomes.

**Results:**

Twenty-four studies were included with 210 patients in the bevacizumab group and 337 patients in the LITT group. Bevacizumab demonstrated symptomatic improvement/stability in 87.7% of cases, radiological improvement/stability in 86.2%, and steroid wean-off in 45%. LITT exhibited symptomatic improvement/stability in 71.2%, radiological improvement/stability in 64.7%, and steroid wean-off in 62.4%. Comparative analysis revealed statistically significant differences favoring bevacizumab in symptomatic improvement/stability (*p* = 0.02), while no significant differences were observed in radiological improvement/stability (*p* = 0.27) or steroid wean-off (*p* = 0.90). The rates of adverse reactions were 11.2% for bevacizumab and 14.9% for LITT (*p* = 0.66), with the majority being grade 2 or lower (72.2% for bevacizumab and 62.5% for LITT).

**Conclusion:**

Both bevacizumab and LITT exhibited favorable clinical and radiological outcomes in managing RN. Bevacizumab was found to be associated with better symptomatic control compared to LITT. Patient-, diagnosis- and lesion-related factors should be considered when choosing the ideal treatment modality for RN to enhance overall patient outcomes.

**Supplementary Information:**

The online version contains supplementary material available at 10.1007/s11060-024-04650-1.

## Introduction

Recent advances in radiation oncology, including stereotactic radiosurgery (SRS), enabled the delivery of higher doses of radiation to the brain with improved precision, making it an important treatment modality for managing primary and metastatic central nervous system (CNS) tumors [1]. This treatment comes with the risk of potential of radiation necrosis (RN), a local inflammatory reaction that can cause significant morbidity and limit its further use. RN typically manifests within 3 to 12 months following the treatment; however, it may develop as late as 3–5 years post-treatment [2, 3]. The incidence of RN has been reported to range from 2 to 46% [2, 4–6], depending on tumor volume, modality, total dose of radiation, and the fractionation [5]. Patients may be asymptomatic or present with symptoms including seizures, memory loss, or signs of increased intracranial pressure, varying with the location and severity of RN [7, 8]. The diagnosis of RN remains challenging, particularly in differentiating it from tumor recurrence. Although there is no definitive radiographic definition for RN, an increase in T2-fluid-attenuated inversion recovery (FLAIR) signal and ‘soap bubble’ or ‘Swiss cheese’ enhancement in T1 on MR imaging support the RN diagnosis [2, 8]. Functional imaging techniques such as fluorodeoxyglucose-positron emission tomography have also been reported to be effective in differentiating RN from tumor recurrence [9]. While these methods along with the review of clinical findings may assist in the interpretation of the radiological findings, a histopathological diagnosis is ultimately required for confirmation.

The underlying pathophysiology of radionecrosis remains unclear. High-dose radiation is thought to disrupt the blood-brain barrier, leading to the development of vasogenic brain edema. This condition compromises the vascular system, ultimately resulting in tissue hypoxia. Tissue hypoxia triggers the release of substances such as VEGF, which stimulates blood vessel growth, further facilitating vasogenic edema [2, 8, 10–12].

Management options of RN include steroids, surgical resection, pentoxifylline, hyperbaric oxygen (HBO), bevacizumab, and laser interstitial thermal therapy (LITT). Steroids are typically the first-line treatment option for patients with new or progressive symptoms related to RN [13]. Steroids reduce cerebral edema and provide resolution of mass-effect-related symptoms by suppressing inflammation [4, 5, 14]. However, long term steroid use is associated with side effects including diabetes mellitus, impaired wound healing, and infections, which may lead to significant morbidity [10]. Surgical resection offers both rapid symptomatic relief and tissue diagnosis, which is particularly critical when there is no clear distinction between radiation necrosis and tumor progression-recurrence [2, 8]. This invasive approach may not be suitable for patients with poor clinical status or for lesions that are not amenable to surgery [8]. Pentoxifylline and HBO have been reported to be effective in treating RN, although supporting evidence for their therapeutic benefit remains weak [3, 15, 16].

Bevacizumab, an anti-VEGF antibody, is the only treatment modality that has been demonstrated to be effective for treating RN in a randomized controlled trial [17]. Bevacizumab mitigates the pathological cascade that leads to vasogenic edema by inhibiting angiogenesis and decreasing vessel permeability [8]. On the other hand, LITT, a minimally invasive surgical option, limits further vasogenic edema by delivering intralesional heat around the periphery of the laser catheter, ablating dysfunctional endothelial cells and astrocytes, which are the origin of VEGF [3, 8].

In this study, we aim to present current evidence on the efficacy of bevacizumab and LITT for treating RN in previously radiated CNS neoplasms and assess differences in outcomes including symptomatic and radiological improvement, as well as steroid wean-off.

## Methods

### Literature review

A systematic review was performed following the Preferred Reporting Items for Systematic Reviews and Meta-Analyses (PRISMA) guidelines [18]. PubMed, Cochrane, Scopus, and EMBASE databases were screened for eligible articles using the following search string: (radiation necrosis OR radionecrosis OR cerebral radionecrosis) AND (bevacizumab OR laser interstitial thermal therapy OR LITT). References of eligible studies were also screened for additional relevant literature. Detailed search strategy is provided in Supplementary File 1.

### Study selection

Inclusion criteria were defined using The Population, Intervention, Control, Outcomes, and Study Design (PICOS) method. Retrieved studies were included if (i) they were retrospective or prospective studies with at least 5 patients with radiation necrosis (Study design) who were previously treated with radiotherapy for primary or secondary CNS tumors (Population), (ii) the treatment for radiation necrosis was bevacizumab or LITT (Intervention, Comparison), (iii) sufficient data on symptomatic control, radiological control, and steroid wean-off were available (Outcome). Systematic reviews and meta-analyses, laboratory or animal studies, studies not written in English, and studies with an unclear distinction between radiation necrosis and other CNS pathologies, or with insufficient data on clinical outcomes were excluded.

Three authors independently screened titles and abstracts of retrieved studies and reviewed the full texts of studies that met the inclusion criteria. Disagreements were resolved by a fourth author.

### Data extraction

The following data were collected from the articles: authors, year, study design, cohort size, age, gender, primary diagnosis, radiotherapy modality, diagnostic modality of radiation necrosis, number of patients with symptoms, number of patients treated with steroids, bevacizumab dosage and cycles, length of hospitalization after LITT, adverse reactions, radiological improvement/stability, symptomatic improvement/stability, and steroid wean-off.

Outcome measures were the proportions of patients with symptomatic and radiologic control and the proportion of patients who were able to wean-off steroids following the treatment. Due to the inconsistencies in outcome reporting among studies, symptomatic control was defined as patients experiencing symptomatic improvement/stability, while radiologic control was defined as patients achieving radiologic improvement/stability after treatment.

Data were extracted by a single author, and independently verified by two authors.

### Risk of bias assessment

Risk of bias assessment was performed using the Joanna Briggs Institute (JBI) checklists for case series and randomized controlled trials [19].

### Statistical analysis

Categorical variables are presented as percentages and continuous variables are presented as means or medians and ranges. Two-sample weighted means t-test was conducted to compare mean pre-treatment RN volumes. Indirect meta-analysis with random effect modeling was performed for radiological responses, symptomatic improvement, weaning-off steroids, adverse events. Outcomes were shown as pooled proportions of events. The Freeman-Tukey transformation was applied to include studies with 0 or 1 event rates and to stabilize variance [20]. Additionally, the DerSimonian-Laird approach for random-effects models was employed to address the high variability observed between studies [21]. I^2^% signifies the heterogeneity between studies. Studies with an I^2^ > 75% were considered to have high heterogeneity. A *p*-value < 0.05 was considered statistically significant. Statistical analyses were performed using SPSS 23.0 (IBM, New York) and RStudio Version 2023.09.1 + 494.

## Results

**Fig. 1:**
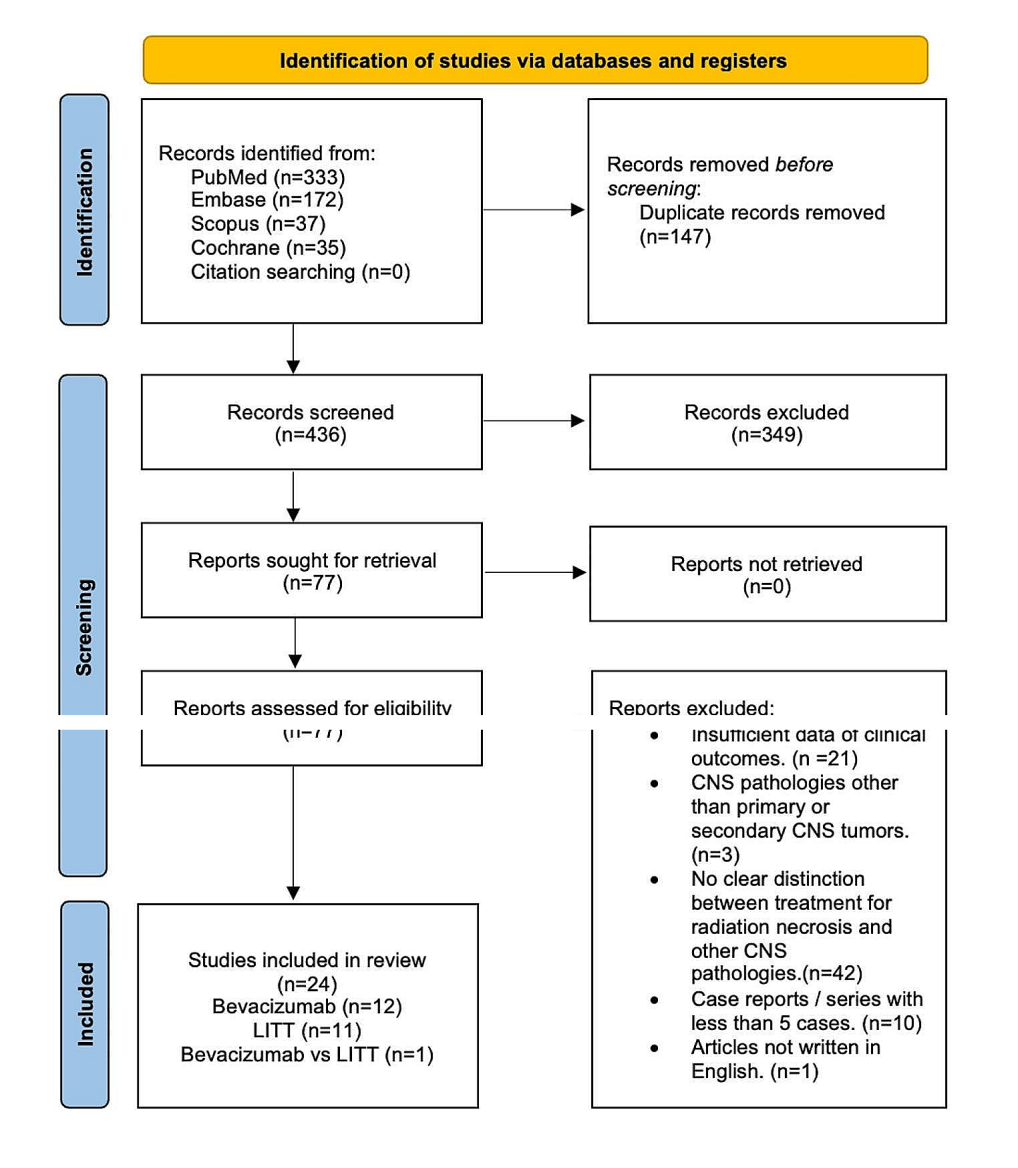
PRISMA flow diagram

PRISMA flow diagram of the literature search and study selection was demonstrated in Fig. 1. Literature search yielded 426 citations after removing duplications. 24 of these citations were identified as eligible and included in the study according to inclusion criteria (Supplementary File 2) [10, 22–44].

### Bevacizumab

Thirteen studies with a total of 210 patients were identified in the literature search [10, 22–33]. A summary of the included studies was shown in Table 1. Median age was 55 years (1.2–76) with 41.8% of the patients being female. 69.5% of the patients had brain metastasis and 30.5% of the patients had a primary CNS tumor and received radiotherapy. Primary CNS tumor diagnoses included glioblastoma multiforme (GBM) (11.4%), diffuse intrinsic pontine glioma (3.8%), high-grade glioma (1.9%), low-grade glioma (1.9%), ependymoma (1.9%), anaplastic oligodendroglioma (1.4%), medulloblastoma (1.4%), anaplastic meningioma (1.4%), anaplastic astrocytoma (1%), anaplastic ependymoma (0.5%), chordoma (0.5%), craniopharyngioma (0.5%), atypical teratoid-rhabdoid tumor (0.5%), tectal astrocytoma (0.5%), anaplastic oligoastrocytoma (0.5%), and hemangiopericytoma (0.5%). 49.8% of the patients had undergone stereotactic radiosurgery/radiotherapy (SRS/SRT) only, 25.9% received other radiotherapy modalities along with SRS/SRT including whole-brain radiotherapy (WBRT), intensity-modulated radiation therapy (IMRT), fractionated/hypofractionated radiotherapy, external beam radiation therapy (EBRT), and proton beam therapy, and 25.9% received only other RT modalities.

Diagnosis of RN was made by radiological images in the majority of the studies (98.6%) with biopsy being the diagnostic modality only in three (1.4%) cases. Pre-treatment RN volumes were reported in seven studies, with a mean of 26.5 cm^3^ (0.1–151). In twelve studies with available data, 96.5% of the patients presented with symptoms. Eight studies reported steroid use with 90.7% of the patients receiving palliative steroids before beginning treatment with bevacizumab. Boothe et al. mentioned that two of their patients were not administered steroids due to extensive metastatic disease. Baroni et al. reported that most of their patients had used steroids but did not provide individual data. Additionally, Furuse et al. and Yonezewa et al. noted that all patients in their studies received conventional medical treatments before starting bevacizumab therapy, although specific details were not provided [30, 33]. Patients received bevacizumab for a median of 4 cycles (1–31) with most common dosing schemes being as the following: 5 mg/kg, every 2 weeks (34.4%), 10 mg/kg, every 2 weeks (17.5%), 7.5 mg/kg, every 3 weeks (11.1%), and 1 mg/kg, every 3 weeks (11.1%) (Table 1).

In twelve studies with available data, symptomatic improvement/stability was observed in 87.7% of cases (Table 1). Radiological improvement/stability was achieved in 86.2% of patients. Among the five studies with available data, 46.6% of patients were reported to have successfully weaned off steroids.

Data regarding adverse reactions were reported in twelve studies. The reporting of adverse reactions was inconsistent among studies, with the overall adverse reaction rate being 11.9%. Of the seven studies that reported adverse reactions using the “Common Terminology Criteria for Adverse Events, v5.0”, rates of grade 1, 2, 3 and 4 reactions were 44.4%, 27.8%, 22.2% and 5.6%, respectively.

**Table 1:** Summary of cohort demographics, clinical characteristics, and outcomes

Characteristics*	**Bevacizumab**	**LITT**	***p*** Value** (I ^**2**^ **%)**
Total of patients	210	337	
Median age, years (range)	55 (1.2–76)	60.7 (23–84)	
Gender, female	41.8%	62.8%	0.01 (58%)
Initial diagnosis			0.21 (95%)
Primary CNS tumor	30.5%	9.6%	
Secondary CNS tumor	69.5%	90.4%	
History of radiotherapy			
Stereotactic radiotherapy/radiosurgery (SRT/SRS) only	49.8%	78.6%	0.07 (88%)
**SRT/SRS + other RT modalities**	24.4%	16.7%	0.63 (79%)
Other RT modalities only	25.9%	4.7%	0.06 (91%)
RN diagnostic modality			
Radiological diagnosis only	207 (98.6)	14 (4.2)	
Biopsy ± radiological diagnosis	3 (1.4)	323 (95.8)	
Patients with symptoms	96.5%	57.4%	0.05 (91%)
Mean pre-treatment RN volume, cm^3^ (range)	26.5 (0.1–151)	4.76 (0.25–31.37)	0.09^a^
Treatment with steroids	90.7%	71.2%	0.05 (90%)
Bevacizumab			
Median cycles (range)	4 (1–31)	Not applicable	
Dosage			
5 mg/kg, q2wks	34.4%	Not applicable	
10 mg/kg, q2wks	17.5%	Not applicable	
7.5 mg/kg, q3wks	11.1%	Not applicable	
1 mg/kg, q3wks	11.1%	Not applicable	
7.5 mg/kg, q2wks	10.6%	Not applicable	
5 mg/kg, q3-4wks	7.4%	Not applicable	
10 mg/kg, q3wks	4.8%	Not applicable	
15 mg/kg, q3wks	2.1%	Not applicable	
15 mg/kg, q4wks	0.5%	Not applicable	
15 mg/kg, q6wks	0.5%	Not applicable	
LITT			
Mean hospital stay, days (range)	Not applicable	1.7 (0.5–6.5)	
Radiological improvement/stability	86.2%	64.7	0.27 (90%)
Post-treatment symptomatic improvement/stability	87.7%	71.2%	0.02 (70%)
Post-treatment steroid wean-off	45%	62.4%	0.90 (81%)
Adverse events	11.9%	14.3%	0.66 (59%)
Grade 1	44.4%	25%	
Grade 2	27.8%	37.5%	
Grade 3	22.2%	25%	
Grade 4	5.6%	12.5%	

*Patients with available data, **The *p*-values are derived from the test for subgroup differences using random effect modeling, and the I^2^% signifies the heterogeneity between studies. ^a^Two-sample weighted means t-test.

### LITT

Twelve studies with a total of 337 patients were identified in the literature search [10, 34–44]. A summary of the included studies was shown in Table 1. Median age was 60.7 years (23–84). 62.8% of the patients were female. Brain metastases were the main initial diagnosis (90.4%) among the included studies with 9.6% of the cases involving primary CNS tumors. Primary CNS tumors included GBM (3%), anaplastic astrocytoma (2.1%), anaplastic oligodendroglioma (0.6%), anaplastic oligoastrocytoma (0.6%), atypical meningioma (0.3%), oligodendroglioma (*n* = 1, 0.3%), esthesioneuroblastoma (0.3%), and malignant intracranial peripheral nerve sheath tumor (0.3%). 78.6% of the patients underwent SRS/SRT only, 16.7% received other radiotherapy modalities along with SRS/SRT such as WBRT, IMRT, and EBRT, and 4.7% received other RT modalities only.

Biopsy was the primary method for diagnosing radiation necrosis (95.8%), with only one study conducted by Rao et al., consisting of 14 cases (22.7%), relying solely on radiological images for diagnosis [37]. Nine studies reported a pre-treatment volume of RN, and the average volume was 4.76 cm^3^ (0.25–31.37). Among the seven studies with available data, 57.4% of the patients were symptomatic. Nine studies reported steroid use prior to treatment, with 71.2% of the patients were using steroids.

Among the five studies analyzed, 71.2% of the patients had symptomatic improvement/stability (Table 1) after LITT treatment. In the nine studies with available data, radiological improvement/stability was achieved in 64.7% of patients. 62.4% of the patients (from six studies) were able to wean-off steroids following the LITT treatment.

Data regarding adverse reactions were reported in eleven studies. Similar to the bevacizumab group, the reporting of adverse reactions was inconsistent among studies, with the overall adverse reaction rate being 14.3%. Of the four studies that reported adverse reactions using the “Common Terminology Criteria for Adverse Events, v5.0”, rates of grade 1, 2, 3 and 4 reactions were 25%, 37.5%, 25% and 12.5%, respectively.

### Comparison of clinical and radiological outcomes

**Fig. 2:**
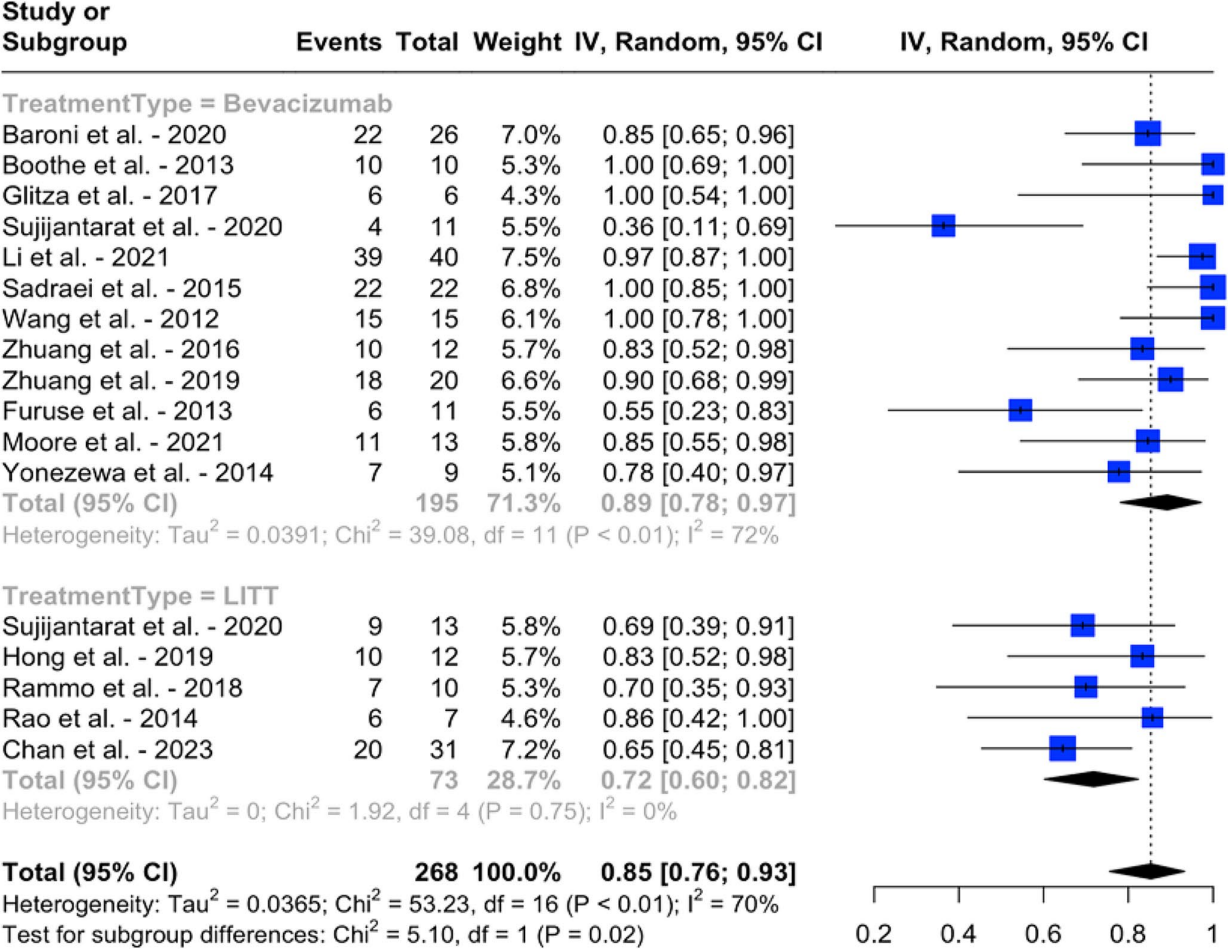
Pooled proportions of symptomatic improvement/stability with bevacizumab and LITT, and results of an indirect meta-analysis comparing the two treatment modalities

**Fig. 3:**
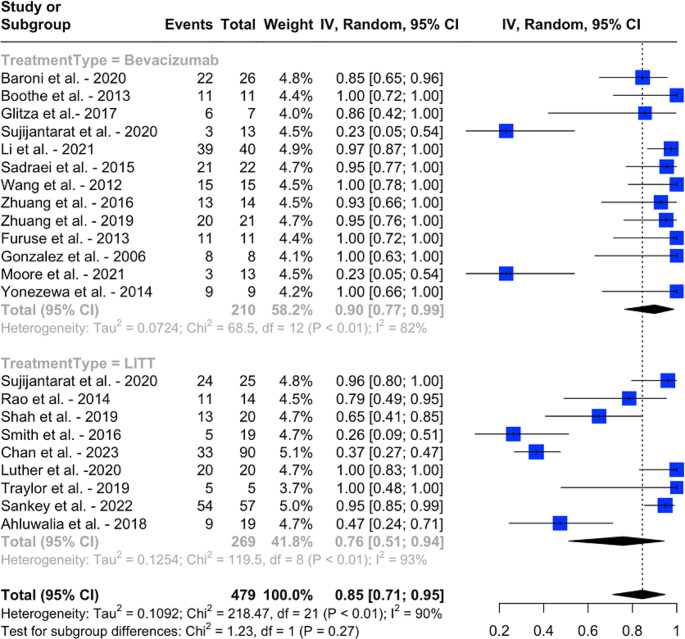
Pooled proportions of radiological improvement/stability with bevacizumab and LITT, and results of an indirect meta-analysis comparing the two treatment modalities

**Fig. 4:**
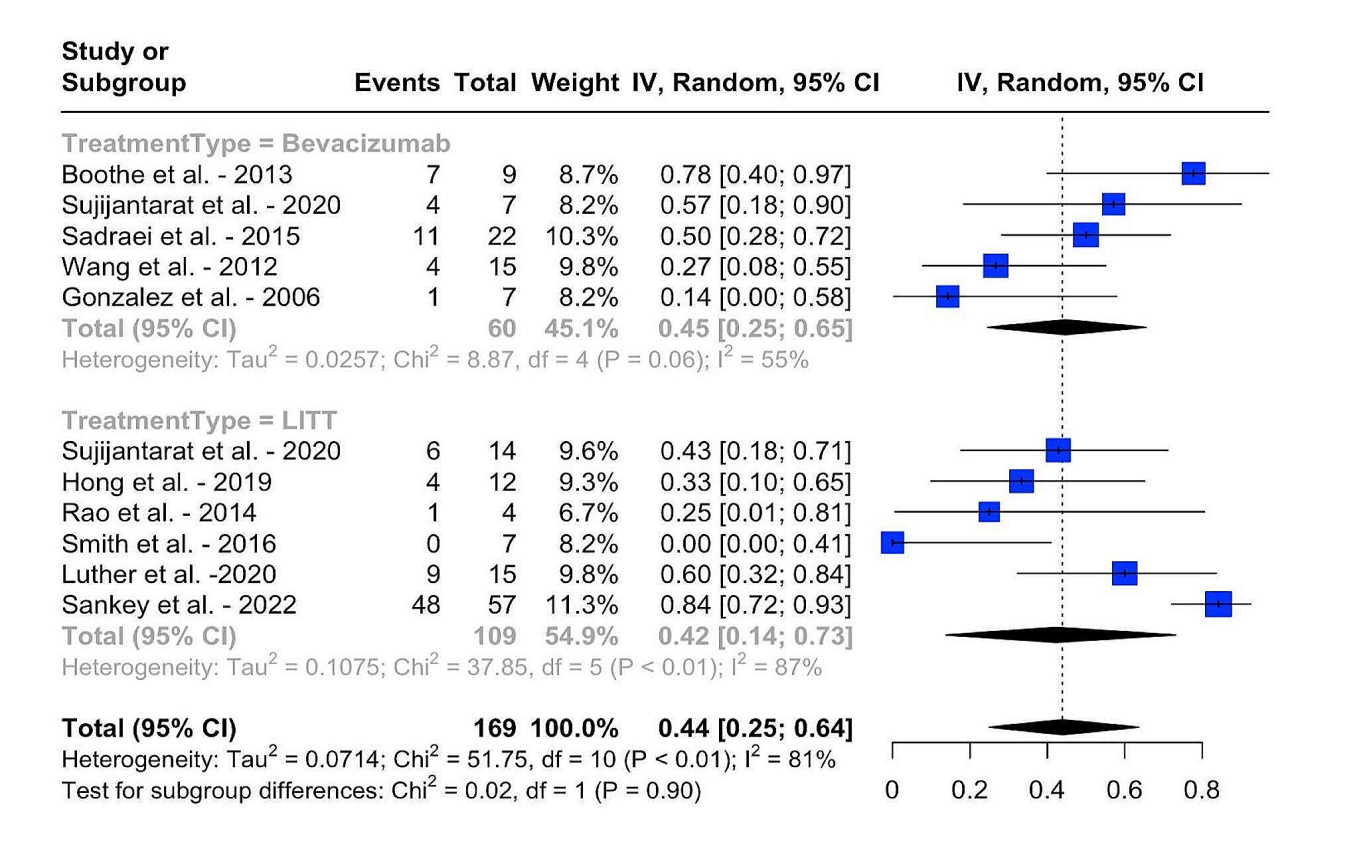
Pooled proportions of patients who were able to wean-off steroids following the treatment with bevacizumab and LITT, and results of an indirect meta-analysis comparing the two treatment modalities

In the bevacizumab group, the pooled proportion of patients achieving symptomatic improvement/stability was 89% (95% CI: 78 − 97%, I^2^ = 72%), compared to 72% (95% CI: 60 − 82%, I^2^ = 0%) in the LITT group, with a statistically significant difference (*p* = 0.02, I2 = 70%) (Fig. 2). Similarly, in terms of radiologic improvement/stability, the pooled rate was 90% (95% CI: 77 − 99%, I2 = 82%) for the bevacizumab group and 76% (95% CI: 51 − 94%, I^2^ = 93%) for the LITT group, with no clinically significant difference observed (*p* = 0.27, I^2^ = 90%) (Fig. 3). The pooled proportion of patients who were able to wean off steroids was 45% (95 CI: 25 − 65%, I^2^. = 55%) in the bevacizumab cohort and 42% (95 CI: 14 − 73%, I^2^. = 87%) in the LITT cohort, with the difference not being statistically significant (*p* = 0.90, I^2^ = 81%) (Fig. 4).

### Quality assessment

The JBI criteria-based assessment for risk of bias revealed that all the studies included had a low risk of bias (Supplementary File 3).

## Discussion

RN is a localized inflammatory reaction that arises in response to radiation injury, often manifesting months to years following radiotherapy. Radiation triggers vasogenic edema and hypoxia in the brain, causing the release of hypoxia-inducible factor 1α (HIF1α) [4, 5, 45]. HIF1α induces the expression of VEGF, which increases vessel permeability and disrupts blood-brain barrier, further propagating the vasogenic edema [4, 5, 45]. The reported incidence of RN ranges between 2% and 25% and may occur in up to 46% of patients following stereotactic radiosurgery/radiotherapy [3, 5, 6]. In this meta-analysis, the majority of patients in both groups primarily underwent stereotactic radiotherapy and radiosurgery for their primary diagnosis (72.4% and 95.3% respectively). It is noteworthy that while the difference between the percentages of patients undergoing SRS is significant (*p* = 0.04), this finding is limited by significant heterogeneity (82%).

Patients with RN often present with a range of symptoms including confusion, seizures, motor weakness and gait disturbance depending on the size and the location of the lesion [5]. Conversely, these symptoms may be subtle in some of the cases, and patients may require further detailed assessments [5]. Among the included studies in this meta-analysis, 96.5% of the patients in the bevacizumab group and 57.4% of the patients in the LITT group were symptomatic (*p* = 0.05, I^2^ = 91%). This difference may be attributed to the fact that among some studies in the LITT group, although individual data was not provided, there were asymptomatic patients in their cohorts who underwent LITT for biopsy-proven RN due to progression on imaging during follow-up, necessitating a tissue diagnosis. Similarly, the mean pre-treatment RN volume was much smaller in the LITT group compared to bevacizumab (4.76 cm^3^ vs. 26.5 cm^3^), which may also explain the disparity in symptomatic patient rates between the two groups. Although this striking difference did not reach statistical significance (*p* = 0.09), presumably due to the limited data, the perilesional edema following LITT treatment, which is typically more prevalent and more severe with larger lesions and sometimes poorly tolerated by patients, may explain this observed trend [46].

Radiological diagnosis of RN is challenging, particularly in differentiating it from recurrence. A histopathological examination may be pursued when feasible for confirmation of the diagnosis. Among the studies included in this meta-analysis, RN was diagnosed through the evaluation of MRI scans in 98.6% of the patients in the bevacizumab group, while biopsy was the primary diagnostic modality in the LITT cohort (95.8%). Biopsy is typically performed in the same session as thermal ablation during LITT; however, it is generally avoided prior to the initiation of bevacizumab treatment due to the risk of intracerebral hemorrhage and concerns regarding wound healing [47].

The majority of the patients in both groups were on palliative steroids before treatment with bevacizumab or LITT (90.7% and 71.2%). Steroids mitigate cerebral edema by suppressing pro-inflammatory reactions, reducing radiation-induced cytokine response, and improving the blood-brain barrier function [5, 45]. Steroids are typically used as a first line treatment in patients who have new-onset or progressive symptoms, and a rapid symptomatic relief is usually observed compared to tumor recurrence [13]. However, some patients require prolonged use of steroids and long-term steroid use is associated with various adverse reactions including steroid dependency, myopathy, osteopenia, and infections, rendering this approach unsustainable [5].

Bevacizumab and LITT are both viable treatment options for RN. Bevacizumab remains to be the only treatment modality that has Level I evidence supporting its efficacy [17]. Levin et al. conducted a randomized, double-blind controlled trial with 14 patients (This study did not meet our inclusion criteria because there were < 5 patients with CNS tumors treated with bevacizumab) with head/neck cancers and CNS tumors, and all patients treated with bevacizumab demonstrated symptomatic and radiological improvement [17]. It mitigates the pathological cascade that leads to vasogenic edema by binding VEGF, decreasing vessel permeability. LITT, a minimally invasive approach that has gained prominence in neurosurgery, particularly for managing deep-seated or inaccessible lesions, ablates the perinecrotic area of gliosis, which harbors most of the astrocytes and endothelial cells that are the origin of VEGF [4, 38].

In this meta-analysis, both bevacizumab and LITT have been demonstrated to have a positive impact on clinical and radiological outcomes following the treatment for RN. The majority of patients in both groups achieved symptomatic improvement/stability (87.7% for bevacizumab and 71.2% for LITT, *p* = 0.02, I^2^ = 70%). The observed difference between the two treatment modalities can be explained by the difference in their mechanism of action. The properties of bevacizumab, such as decreasing vessel permeability, which disrupt the cascade that worsens vasogenic edema, may play a role in relieving the mass effect upfront and thus improving and/or controlling symptoms [10]. Additionally, the relatively higher rate of steroid use in the bevacizumab group (90.7% versus 71.2% in the LITT group) may have played a role in higher symptomatic improvement/stability rate in this group, although the difference is limited by significant heterogeneity (*p* = 0.05, I^2^ = 90%).

Radiological improvement/stability was reported in 86.2% of the patients in the bevacizumab group and 64.7% in the LITT group (*p* = 0.27, I^2^ = 90%). Although not significant and limited due to significant heterogeneity, this difference between groups may be explained by inconsistencies in reporting radiological outcome among studies in the LITT group. The time-point at which radiological response was assessed following treatment with LITT varied between 2 months to 1 year among studies. LITT may result in an initial increase in the lesion volume due to perilesional edema and an expanding necrotic area around the RN, giving a false impression of disease progression. However, resolution or volume reduction of the lesion is typically observed during long-term follow-up, usually within 12–15 months post-treatment [2, 10, 48]. It is pertinent to note that Palmisciano et al. reported that bevacizumab was superior to LITT with concerning partial radiological response (79.6% versus 29.5%, *p* = 0.001, I^2^ = 88.9%) in patients with RN who received RT due to brain metastases [8]. However, when patients with complete and partial response and stable disease were combined, these rates were similar (89.8% versus 86.9%) [8]. Our radiological control rates differed compared to that study as we included more studies and defined our outcome as radiological improvement/stability. Similarly, Vellayappan et al. recently conducted a systematic review regarding the management of symptomatic RN following SRS and reported similar radiological improvement/stability rates (93% for bevacizumab, 88% for LITT) for both groups [49]. Our findings again differed from that study numerically as we included both symptomatic and asymptomatic patients, as well as patients underwent other RT modalities.

Both bevacizumab and LITT led to comparable results in terms of weaning of steroids (45% in bevacizumab and 62.45% in LITT, *p* = 0.90, I^2^ = 81). It is pertinent to note that the clinical status of each patient varied between studies, inherently affecting the decision to wean off steroids, and may explain the high heterogeneity observed among studies. Both bevacizumab and LITT showed favorable safety profiles with rates of adverse events of 11.2% and 14.9% (*p* = 0.66, I^2^ = 59%). Although sporadic serious adverse reactions including thromboembolic events and intracerebral hemorrhage occurred in both cohorts, most of these reactions were grade 2 or less [26, 32, 36, 37].

In summary, in this meta-analysis, both bevacizumab and LITT resulted in favorable clinical outcomes. Bevacizumab has been found to be associated with better symptomatic control. While further prospective studies and randomized-controlled trials will help validate this result, bevacizumab may be highly considered in patients with symptoms that do not require immediate surgical intervention due to its efficacy and favorable safety profile. LITT led to comparable outcomes to bevacizumab with much smaller lesion volumes. While further research is needed to assess its efficacy in larger lesions, LITT offers the advantage of providing a histopathological diagnosis and can be a more viable option when tissue diagnosis is required. Additionally, it may serve as an alternative treatment for patients who have not responded to steroids and bevacizumab therapy [49]. In all cases, patient-, diagnosis- and lesion-related factors should be at the center of decision-making when choosing a treatment modality.

### Limitations

This study has several limitations. Only a few studies reported individual patient data; most of the information was derived from diverse patient populations, limiting our ability to perform subgroup analyses. Data regarding radiological and clinical outcomes were limited in some studies and unevenly distributed among treatment groups, making it challenging to reliably compare the two treatment modalities. Furthermore, except for the three prospective studies, the majority of the included studies were retrospective, which inherently carries the risk of selection and recall bias, rendering it difficult to draw robust conclusions.

## Conclusion

We presented the current evidence on the efficacy of bevacizumab and LITT for treating RN in previously radiated CNS neoplasms and compared the clinical and radiological outcomes associated with their use. Both treatment modalities led to comparable results with respect to radiological control and steroid wean-off, with favorable toxicity profiles. Bevacizumab was found to be associated with better symptomatic control; however, further research is required to validate this result. Our study results underscore the importance of considering the patient status and lesion characteristics when choosing the treatment modality for RN to enhance overall patient outcomes.

### Electronic supplementary material

Below is the link to the electronic supplementary material.

Supplementary Material 1

Supplementary Material 2

Supplementary Material 3

## Data Availability

No datasets were generated or analysed during the current study.

## Abbreviations

RN: radiation necrosis
LITT: laser interstitial thermal therapy
CNS: central nervous system
SRS: stereotactic radiosurgery
VEGF: vascular endothelial growth factor
HIF1α: hypoxia-inducible factor 1α, WBRT:whole-brain radiotherapy
IMRT: intensity-modulated radiation therapy
EBRT: external-beam radiation therapy
GBM: glioblastoma multiforme
