# Supplementary material for: Comparative analysis of bevacizumab and LITT for treating radiation necrosis in previously radiated CNS neoplasms: a systematic review and meta-analysis
Source: J Neurooncol. 2024 Apr 15;168(1):1–11. doi: 10.1007/s11060-024-04650-1 (PMC11093788; doi:10.1007/s11060-024-04650-1)
Supplement: Supplementary file 1 — Supplementary Material 1 [file 11060_2024_4650_MOESM1_ESM.docx]

| Database | Search strategy | Number |
| --- | --- | --- |
| PubMed | #1 (“radionecrosis”[tiab] OR “radiation necrosis”[tiab] OR “cerebral radionecrosis”[tiab])  #2 ((“Bevacizumab”[Mesh] OR "Bevacizumab”[tiab] OR “anti-vegf”[tiab] OR “avastin”[tiab] OR “vegf”[tiab] OR “antiangiogenic”[tiab]) OR (“laser interstitial thermal therapy”[tiab] OR “LITT”[tiab]))  #3 #1 AND #2 AND #3 | **333** |
| Embase | #1 (“'radionecrosis”:ti,ab OR “radiation necrosis”:ti,ab OR “cerebral radionecrosis”:ti,ab)  #2 (('anti-vegf':ti,ab OR 'avastin':ti,ab OR 'vegf':ti,ab OR 'bevacizumab'/exp OR 'antiangiogenic':ti,ab) OR (“laser interstitial thermal therapy”:ti,ab OR “LITT”:ti,ab))  #3 #1 AND #2 | **172** |
| Scopus | #1 TITLE-ABS-KEY (“radionecrosis” OR “radiation necrosis” OR “cerebral radionecrosis)  #2 TITLE-ABS-KEY ((“Bevacizumab*” OR “anti-vegf” OR “avastin” OR “vegf”) OR (“laser interstitial thermal therapy” OR “LITT”))  #3 #1 AND #2 | **37** |
| Cochrane | #1 ("radionecrosis" OR “radiation necrosis” OR "cerebral radionecrosis"):ti,ab,kw  #2 (("Bevacizumab”[Mesh] OR “anti-vegf” OR “avastin” OR “vegf”):ti,ab,kw OR (“laser interstitial thermal therapy” OR “LITT”):ti,ab,kw)  #3 #1 AND #2 | **35** |
