# Supplementary material for: Comparative analysis of bevacizumab and LITT for treating radiation necrosis in previously radiated CNS neoplasms: a systematic review and meta-analysis
Source: J Neurooncol. 2024 Apr 15;168(1):1–11. doi: 10.1007/s11060-024-04650-1 (PMC11093788; doi:10.1007/s11060-024-04650-1)
Supplement: Supplementary file 2 — Supplementary Material 2 [file 11060_2024_4650_MOESM2_ESM.docx]

| Table 1. Overview of included studies for Bevacizumab | | | | | | | | | | | | | | | | |
| --- | --- | --- | --- | --- | --- | --- | --- | --- | --- | --- | --- | --- | --- | --- | --- | --- |
| Authors-Year | **Study Design** | **Patients No./ Female** | **Age-years median (range)** | **Primary Dx No. patients (%) (primary vs secondary)** | **RT modality No. patients (%)** | **Patients with RN symptoms No. patients (%)** | **Diagnosis of RN (Imaging vs Bx) No. patients (%)** | **Total no. RN lesions / Mean no. RN lesions per patient** | **Mean pre-treatment RN volume (cm3) (range)** | **Steroids use No. patients (%)** | **Bevacizumab dosage No. patients (%)** | **Bevacizumab cycles median (range)** | **Bevacizumab Adverse Events  No. patients (%) No. per grade** | **Post-treatment symptomatic control No. patients (%)** | **Post-treatment weaning off steroids No. patients (%)** | **Post-treatment radiological control No. patients (%)** |
| Baroni et al^22^ | Retrospective, multicenter | 26 / NR | 10,7 (1,4 - 18,3) | 26 (100) primary CNS tumor | 22 IMRT (84.6), 4 (15.4) Proton therapy | 26 (100) | Imaging: 26 (100) | NR | NR | NR | 10 mg/kg, q2wks: 18 (69.2), 7.5 mg/kg, q2wks: 5 (19.2), 5 mg/kg, q2wks: 3 (11.5) | 4.5 (2 - 7) | 1 (3.8): Grade 3 | 22 (84.6) | NR | 22 (84.6) |
| Boothe et al^23^ | Retrospective, single center | 11 / 7 (63.6) | 63 (27 - 79) | 11 (100) secondary CNS tumor | 5 WBRT (45.5), 11 SRT (100) | 10 (90.9) | Imaging: 9 (81.8), Bx: 2 (18.2) | 14 / 1,2 | 69.9 (21.2 – 168.5) | 9 (81.8) | 7.5 mg/kg, q3wks: 1 (9.1), 10mg/kg, q2 wks: 8 (72.7), 15mg/kg, q6wks: 1 (9.1), 15mg/kg, q4wks: 1 (9.1) | 2 (1 - 31) | 0 (0) | 10 (100) | 7 (77.8) | 11 (100) |
| Glitza et al^24^ | Retrospective, single center | 7 / 2 (28.5) | 56 (37 - 71) | 7 (100) secondary CNS tumor | 2 WBRT (28.6), 5 SRT (71.4) | 6 (85.7) | Imaging:7 (100) | NR | NR | 5 (71.4) | 7.5 mg/kg: 5 (71.4), 5 mg/kg: 1 (14.3), 10 mg/kg: 1 (14.3) | 4 (2 - 6) | 1 (14.2): Grade 2 | 6 (100) | NR | 6 (85.7) |
| Sujijantarat et al^10^ | Retrospective, single center | 13 / 3 (23.1) | 63 (33 - 72) | 13 (100) secondary CNS tumor | 13 SRT (100) 2 WBRT (15.4) | 11 (84,6) | Imaging:13 (100) | NR | 3,1 (0,1 - 9,1) | 7 (53.8) | Median 15 mg/kg q4wks (7.5mg/kg – 15mg/kg) | 4 (1 - 7) | 4 (30,8): 2 Grade 1, 1 Grade 2, 1 Grade 3 | 4 (36.4) | 4 (57,1) | 3 (23.1) |
| Li et al^25^ | Retrospective, single center | 40 / 14 (35) | 55,5 (29 - 72) | 40 (100) secondary CNS tumor | 40 SRT (100), 15 WBRT (37,5) | 40 (100) | Imaging: 40 (100) | NR | 18,7 (0,89 - 53,3) | 39 (97,5) | 5 mg/kg, q2wks: 31 (77,5), 10 mg/kg, q3wks: 9 (22,5) | NR | 0 (0) | 39 (97.5) | NR | 39 (97.5) |
| Sadraei et al^26^ | Retrospective, single center | 22 / 13 (59) | 58 (31 - 67) | 5 (22,8) primary, 17 (77,8) secondary CNS tumor | 19 SRT (86,3), 11 WBRT (50), 1 Proton beam (4,54), 3 Fractionated RT (13,6) | 22 (100) | Imaging: 22 (100) | NR | 7,14 (1,3 - 28,1) | 20 (90,9) | 5 mg/kg, q2wks: 5 (22,7), 7.5 mg/kg, q3wks: 5 (22,7), 10 mg/kg, q2wks: 7 (31,8) 15 mg/kg, q3wks: 4 (18,1) | 4 (2 - 13) | 6 (27,2): 1 grade 3, 2 grade 1, 3 grade 2 | 22 (100)) | 11 (55) | 21 (95.5) |
| Wang et al^27^ | Retrospective, single center | 15 / 4 (26,6) | 52 (13 - 71) | 10 (66,7) primary, 5 (33,3) secondary CNS tumor | 4 SRT (26.7), 11 EBRT (73.3), 3 Fractionated RT (20) | 15 (100) | Imaging: 15 (100) | NR | NR | 15 (100) | 7.5 mg/kg, q3wks: 15 (100) | NR | 3 patients experienced adverse events of grade 2 or less-no individual data | 15 (100) | 4 (26,6) | 15 (100) |
| Zhuang et al^28^ | Retrospective, single center | 14 / 8 (57.1) | 53 (31 - 70) | 14 (100) secondary CNS tumor | 14 SRT (100), 4 WBRT (28,6) | 12 (85,7) | Imaging: 13 (92,8), Bx: 1 (7,1) | 14 / 1,0 | NR | NR | 5 mg/kg, q3-4wks: 14 (100) | 3 (3 - 10) | 2 (14,3): 2 Grade 1 | 10 (83,3) | NR | 13 (92.9) |
| Zhuang et al^29^ | Prospective, single center | 21/ 10 (47,6) | 55 ( 43 - 70) | 21 (100) secondary CNS tumor | 21 SRT (100), 5 WBRT (23,8) | 20 (95,2) | Imaging: 21 (100) | 21 / 1,0 | NR | NR | 1 mg/kg, q3wks: 21 (100) | 3 (3 - 5) | 2 (9,5): 2 Grade 1 | 18 (90) | NR | 20 (95.2) |
| Furuse et al^30^ | Retrospective, single center | 11 / 5 (45) | 55 (27 - 76) | 8 (72,7) primary, 3 (27,3) secondary CNS tumor | 6 SRT (54.5) | 11 (100) | Imaging: 11 (100) | NR | 71,6 (29,7 - 151) | NR | 5 mg/kg, q2wks: 11 (100) | 5 (3 - 6) | 0 (0) | 6 (54.5) | NR | 11 (100) |
| Gonzalez et al^31^ | Retrospective, single center | 8 / 4 (50) | 52 (32 - 70) | 8 (100) primary CNS tumor | 3 SRT (37,5) , 8 WBRT (100) | NR | Imaging: 8 (100) | NR | 7,09 (4,42 - 10,9) | 7 (87,5) | 5 mg/kg q2wks: 6 (75), 7.5 mg/kg q3wks: 2 (25) | NR | NR | NR | 1 (14,2) | 8 (100) |
| Moore et al^32^ | Retrospective, multicenter | 13 / 5 (38,4) | 58 (49 - 70) | 13 (100) secondary CNS tumor | 13 SRT (100) | 13 (100) | Imaging: 13 (100) | NR | 7,9 (2,4 - 24,6) | 13 (100) | 7.5 mg/kg q3wks: 13 (100) | 2 (1 - 5) | 2 (15,3): 1 Grade 3, 1 Grade 4 | 11 (84,6) | NR | 3 (32.1)* |
| Yonezewa et al^33^ | Retrospective, single center | 9 / 2 (22,2) | 54 (37 - 64) | 7 (77,7) primary, 2 (22,3) secondary CNS tumor | 3 SRT (33,3), 6 Hypofractionated RT (66,7), 1 WBRT (11,1) | 9 (100) | Imaging: 9 (100) | NR | NR | NR | 5 mg/kg q2wks: 9 (100) | 6 | 3 (33,3): Grade 1 or 2 (not specified) | 7 (77,8) | NR | 9 (100) |
| Abbreviations: Dx, diagnosis; Bx, biopsy; RN, radionecrosis; IMRT, intensity-modulated radiotherapy; WBRT, whole-brain radiotherapy; EBRT, external beam radiotherapy; SRT, stereotactic radiotherapy. *Data was available for nine patients. | | | | | | | | | | | | | | | | |

| Table 2. Overview of included studies for LITT | | | | | | | | | | | | | | | | |
| --- | --- | --- | --- | --- | --- | --- | --- | --- | --- | --- | --- | --- | --- | --- | --- | --- |
| Authors-Year | **Study Design** | **Patients No./ Female** | **Age-years median (range)** | **Primary Dx No. patients (%) (primary vs secondary)** | **RT modality No. patients (%)** | **Patients with RN symptoms No. patients (%)** | **Diagnosis of RN (Imaging vs Bx) No. patients (%)** | **Total no. RN lesions / Mean no. RN lesions per patient** | **Mean pre-treatment RN volume (cm3) (range)** | **Steroids use No. patients (%)** | **LITT System** | **Post-LITT hospital stay  days median (range)** | **LITT Adverse Events  No. patients (%) No. per grade** | **Post-treatment symptomatic improvement No. patients (%)** | **Post-treatment weaning off steroids No. patients (%)** | **RN Radiological Response No. patients (%)** |
| Sujijantarat et al^10^ | Retrospective, single center | 25 / 16 (64) | 62 (35 - 81) | 25 (100) secondary CNS tumor | 25 SRT (100%) 4 WBRT (16%) | 13 (52) | Bx: 25 (100) | NR | 2,2 (0,3 - 12,6) | 14 (56) | NeuroBlate (Monteris Medical Inc., Minneapolis, MN) | 2 (1 - 5) | 3 (12) | 9 (69) | 6 (42,8) | 24 (96%) |
| Hong et al^34^ | Retrospective, single center | 18 / NR | NR | NR | 18 (100) SRT | 12 (66,7) | Imaging: 18 (100), Bx: 18 (100) | NR | NR | 12 (66,7) | NeuroBlate (Monteris Medical Inc., Minneapolis, MN) | 3,1 | 6 (33,3) | 10 (83,3) | 4 (33,3) | NR |
| Kim et al^35^ | Prospective, multicenter | 34 / NR | NR | NR | 34 (100) SRT | NR | NR | NR | NR | NR | NeuroBlate (Monteris Medical Inc., Minneapolis, MN) | 1,5 | 1 (2,2) | NR | NR | NR |
| Rammo et al^36^ | Retrospective, single center | 10 / 6 (60) | 61,5 (44 - 74) | 4 (40) primary, 6 (60) secondary CNS tumor | 10 (100) SRT, 5 (50) EBRT | 10 (100) | Bx: 10 (100) | 10 / 1,0 | 1,62 | 10 (100) | Visualase (Medtronic) | 1,5 | 2 (20): 1 Grade 2, 1 Grade 3 | 7 (70) | NR | NR |
| Rao et al^37^ | Retrospective, single center | 14 / 6 (42,8) | 67 (46 - 82) | 14 (100) secondary CNS tumor | 14 (100) SRT, 5 (35,7) WBRT | 7 (50) | Imaging: 14 (100) | 15 / 1,1 | 3,66 (0,46 - 25,45) | 4 (28,6) | Visualase (Medtronic) | 1,2 (1 - 5) | 2 (14,3): 1 Grade 3, 1 Grade 4 | 6 (85.7) | 1 (25) | 11 (78.8) |
| Shah et al^38^ | Retrospective, single center | 20 / 17 (85) | 60 (46 - 83) | NR | NR | 20 (100) | Imaging: 20 (100), Bx: 20 (100) | 20 / 1,0 | median: 5,9 (0,9 - 31,7) | NR | Visualase (Medtronic) | 1 | 0 (0) | NR | NR | NR |
| Smith et al^39^ | Retrospective, single center | 25 / 15 (60) | 57 (23 - 84) | 18 (72) primary, 7 (28) secondary CNS tumor | 19 (76) SRT, 13 (52) IMRT, 2 (8) WBRT | NR | Bx: 25 (100) | NR | 7,71 (0,25 - 31,37) | 7 (28) | Visualase (Medtronic) | 1 | 2 (8): 1 grade 1, 1 grade 2 | NR | 0 (0) | 5 (26.3) |
| Chan et al^40^ | Retrospective, multi center | 90 / 52 (57,8) | 65 (27 - 83) | 90 (100) secondary CNS tumor | 84 (93,3) SRT, 9 (10) WBRT, 7 (7,8) Local | 31 (34,3) | Bx: 90 (100) | 94 / 1,04 | NR | 72 (80) | NeuroBlate (Monteris Medical Inc., Minneapolis, MN) | 1,3 (1,2 - 2,2) | 17 (18,9) | 20 (64) | NR | 33 (36.7) |
| Luther et al^41^ | Retrospective, single center | 20 / 17 (85) | 60,5 (29 - 83) | 3 (15) primary, 17 (85) secondary CNS tumor | 17 (85) SRT, 5 (25) WBRT, 4 (20) IMRT | 20 (100) | Bx: 20 (100) | NR | 8.474 (0.885 - 31.69) | 15 (75) | Visualase (Medtronic) | 2 (2-3) | 4 (20) | NR | 9 (60) | 20 (100) |
| Traylor et al^42^ | Retrospective, single center | 5 / 5 (100) | 57 (31 - 68) | NR | 5 (100) SRT, 1 (20) WBRT | NR | Imaging: 5 (100), Bx: 5 (100) | NR | 5,26 (1,61 - 8,11) | NR | NeuroBlate (Monteris Medical Inc., Minneapolis, MN) | NR | 1 (20) | NR | NA | 5 (100) |
| Sankey et al^43^ | Retrospective, multi center | 57 / 33 (58) | 59 (55 - 69) | 57 (100) secondary CNS tumor | 57 (100) SRT, 17 (30) WBRT | NR | Imaging: 57 (100), Bx: 57 (100) | NR | 3.70 (2.33-6.05) | 57 (100) | NR | NR | NR | NR | 48 (84) | 54 (94.7) |
| Ahluwalia et al^44^ | Prospective, multicenter | 19 / 12 (63,2) | 58 (49 - 72) | 19 (100) secondary CNS tumor | 19 (100) SRT | NR | Bx: 19 (100) | 19 / 1,0 | 5.5 (0.4–13.2) | 7 (36,8) | NeuroBlate (Monteris Medical Inc., Minneapolis, MN) | 1,7 (0,5 - 6,5) | 2 (10.5): 1 Grade 1 1 Grade 2 | NR | NR | 9 (47.4) |
| Abbreviations: Dx, diagnosis; Bx, biopsy; RN, radionecrosis; IMRT, intensity-modulated radiotherapy; WBRT, whole-brain radiotherapy; EBRT, external beam radiotherapy; SRT, stereotactic radiotherapy. | | | | | | | | | | | | | | | | |
