# Supplementary material for: Comparative analysis of bevacizumab and LITT for treating radiation necrosis in previously radiated CNS neoplasms: a systematic review and meta-analysis
Source: J Neurooncol. 2024 Apr 15;168(1):1–11. doi: 10.1007/s11060-024-04650-1 (PMC11093788; doi:10.1007/s11060-024-04650-1)
Supplement: Supplementary file 3 — Supplementary Material 3 [file 11060_2024_4650_MOESM3_ESM.docx]

| Risk of bias assessments for included studies. |
| --- |
| **JBI Checklist for Case Series – Criteria** |
| 1. Were there clear criteria for inclusion in the case series? |
| 2. Was the condition measured in a standard, reliable way for all participants included in the case series? |
| 3. Were valid methods used for identification of the condition for all participants included in the case series? |
| 4. Did the case series have consecutive inclusion of participants? |
| 5. Did the case series have complete inclusion of participants? |
| 6. Was there clear reporting of the demographics of the participants in the study? |
| 7. Was there clear reporting of clinical information of the participants? |
| 8. Were the outcomes or follow up results of cases clearly reported? |
| 9. Was there clear reporting of the presenting site(s)/clinic(s) demographic information? |
| 10. Was statistical analysis appropriate? |
| **Responses Options**: Yes, No, Unclear, Not Applicable (NA) |
| **Quality Rating**: Poor 0 – 3; Fair 4 – 7; Good 8 – 10 |

| **Study** | **1** | **2** | **3** | **4** | **5** | **6** | **7** | **8** | **9** | **10** | **Appraisal** |
| --- | --- | --- | --- | --- | --- | --- | --- | --- | --- | --- | --- |
| Baroni et al^20^ | Y | Y | Y | Y | Y | Y | Y | N | Y | NA | 8 - Good |
| Boothe et al^21^ | Y | Y | Y | Y | Y | Y | Y | N | N | Y | 8 – Good |
| Glitza et al^22^ | Y | Y | Y | Y | Y | N | Y | Y | N | NA | 7- Fair |
| Sujijantarat et al^8^ | Y | Y | Y | Y | Y | Y | Y | Y | N | Y | 9 - Good |
| Li et al^23^ | Y | Y | Y | Y | Y | N | N | N | N | Y | 6 - Fair |
| Sadraei et al^24^ | Y | Y | Y | Y | Y | Y | Y | Y | N | Y | 9 - Good |
| Wang et al^25^ | Y | Y | Y | Y | Y | Y | Y | Y | Y | NA | 9 – Good |
| Zhuang et al^26^ | Y | Y | Y | Y | Y | N | N | N | N | Y | 6 – Fair |
| Hong et al^32^ | Y | Y | Y | Y | Y | Y | N | N | N | NA | 6 – Fair |
| Kim et al^33^ | Y | Y | Y | Y | Y | N | N | N | N | Y | 6 – Fair |
| Rammo et al^34^ | Y | Y | Y | Y | Y | Y | Y | N | Y | Y | 9 – Good |
| Rao et al^35^ | Y | Y | Y | Y | Y | Y | Y | N | Y | Y | 9 – Good |
| Shah et al^36^ | Y | Y | Y | Y | Y | N | Y | N | N | Y | 7 – Good |
| Smith et al^37^ | Y | Y | Y | Y | Y | Y | Y | N | N | Y | 8 – Good |
| Chan et al^38^ | Y | Y | Y | Y | Y | Y | Y | N | Y | Y | 9 – Good |
| Furuse et al^28^ | Y | Y | Y | Y | Y | Y | Y | Y | N | Y | 9 – Good |
| Gonzalez et al^29^ | Y | Y | Y | Y | Y | Y | Y | N | N | NA | 7 – Fair |
| Moore et al^30^ | Y | Y | Y | Y | Y | Y | N | N | N | Y | 7 – Fair |
| Yonezewa et al^31^ | Y | Y | Y | Y | Y | Y | N | N | N | Y | 7 – Fair |
| Luther et al^39^ | Y | Y | Y | Y | Y | Y | N | N | N | Y | 7 – Fair |
| Traylor et al^40^ | Y | Y | Y | Y | Y | Y | N | N | N | Y | 7 – Fair |
| Sankey et al^41^ | Y | Y | Y | Y | Y | N | N | N | N | Y | 6 – Fair |

| **JBI Checklist for Randomized Controlled Trials – Criteria** |
| --- |
| 1. Was true randomization used for assignment of participants to treatment groups? |
| 2. Was allocation to treatment groups concealed? |
| 3. Were treatment groups similar at the baseline? |
| 4. Were participants blind to treatment assignment? |
| 5. Were those delivering treatment blind to treatment assignment? |
| 6. Were outcomes assessors blind to treatment assignment? |
| 7. Were treatment groups treated identically other than the intervention of interest? |
| 8. Was follow up complete and if not, were differences between groups in terms of their follow up adequately described and analyzed? |
| 9. Were participants analyzed in the groups to which they were randomized? |
| 10. Were outcomes measured in the same way for treatment groups? |
| 11. Were outcomes measured in a reliable way? |
| 12. Was appropriate statistical analysis used? |
| 13. Was the trial design appropriate, and any deviations from the standard RCT design (individual randomization, parallel groups) accounted for in the conduct and analysis of the trial? |
| **Responses Options**: Yes, No, Unclear, Not Applicable (NA) |
| **Quality Rating**: Poor 0 – 4; Fair 5 – 9; Good 10 – 13 |

| **Study** | **1** | **2** | **3** | **4** | **5** | **6** | **7** | **8** | **9** | **10** | **11** | **12** | **13** | **Appraisal** |
| --- | --- | --- | --- | --- | --- | --- | --- | --- | --- | --- | --- | --- | --- | --- |
| Ahluwalia et al^42^ | NA | NA | NA | NA | NA | NA | NA | Y | NA | NA | Y | Y | Y | 4* |
| Zhuang et al^27^ | NA | NA | NA | NA | NA | NA | NA | Y | NA | NA | Y | Y | Y | 4* |
| *** The studies were appraised with “good quality”, and the risk of bias was considered low in regards of their design (prospective study). | | | | | | | | | | | | | | |
